# Induced abortion among female students in higher education institutions in Ethiopia: A systematic review and meta-analysis

**DOI:** 10.1371/journal.pone.0280084

**Published:** 2023-01-20

**Authors:** Bereket Kefale, Yitayish Damtie, Mastewal Arefaynie, Melaku Yalew, Bezawit Adane, Tenagnework Dilnesa, Segenet Zewdie, Yitbarek Wasihun, Metadel Adane

**Affiliations:** 1 Department of Reproductive and Family Health, School of Public Health, College of Medicine and Health Sciences, Wollo University, Dessie, Ethiopia; 2 Department of Epidemiology and Biostatistics, School of Public Health, College of Medicine and Health Sciences, Wollo University, Dessie, Ethiopia; 3 Department of Midwifery, School of Nursing and Midwifery, College of Medicine and Health Sciences, Wollo University, Dessie, Ethiopia; 4 Department of Pharmacy, College of Medicine and Health Sciences, Wollo University, Dessie, Ethiopia; 5 Department of Health Promotion, School of Public Health, College of Medicine and Health Sciences, Wollo University, Dessie, Ethiopia; 6 Department of Environmental Health, College of Medicine and Health Sciences, Wollo University, Dessie, Ethiopia; WCU: Wachemo University, ETHIOPIA

## Abstract

**Background:**

Female students in institutions of higher education are at higher risk of abortion and its consequences. There is no nationally representative data on induced abortion among students in higher education institutions in Ethiopia. Hence, this study aimed to estimate the pooled prevalence of induced abortion among female students in institutions of higher education in Ethiopia.

**Methods:**

This study used a systematic review and meta-analysis of studies conducted from January 1, 2010, to June 30, 2022, in Ethiopia. The Preferred Reporting Items for Systematic Reviews and Meta-Analyses (PRISMA) guidelines were followed. PubMed, Cochrane Library, Hinari, Google Scholar, CINAHL, and Global Health electronic databases were searched. The analysis was performed using STATA 14 software. Heterogeneity and publication bias were assessed using I^2^ statistics and Egger’s test, respectively. Duval and Tweedie’s ‘trim and fill’ method was also performed to adjust the pooled estimate. Forest plots were used to present the pooled prevalence with a 95% confidence interval (CI) of meta-analysis using the random effect model.

**Results:**

This systematic review and meta-analysis included a total of 10 studies and 4656 study participants. The pooled prevalence of induced abortion among female students in institutions of higher education in Ethiopia was 5.06% (95%CI: 2.16, 7.96). The rate of induced abortion was 51 per 1000 women.

**Conclusions:**

The pooled prevalence of induced abortion among female students in institutions of higher education in Ethiopia was high. Thus, concerned bodies should design and implement an effective strategy to realize friendly and non-judgmental family planning and comprehensive abortion care service to curb the problem.

## Introduction

Induced abortion is one of the leading causes of maternal morbidity and mortality [1]. Globally, three out of ten pregnancies, and six out of ten unintended pregnancies, ended in an induced abortion [2]. About 45% of all abortions are unsafe, of which 97% occur in developing countries [3]. An estimated 33 abortions occur each year per 1,000 women aged 15–49 in Sub-Saharan Africa, of which more than three-fourths are unsafe [4]. In Ethiopia, about 28 abortions per 1,000 women aged 15–49 took place in 2014 [5]. A multilevel analysis based on the 2016 Ethiopian Demographic Health Survey (EDHS) also showed that the magnitude of abortion among youths was 2.5% [6]. The magnitude of induced abortion among higher education institution female students in Ethiopia ranged from 2.8% to 18.8% [7, 8].

Unsafe abortion is a serious reproductive health problem that adversely affects women’s physical, mental, and social well-being throughout their lifetime [9–15]. Unsafe abortion accounts for 4.7 to 13.2% of maternal deaths that occur globally [1]. The physical health complications caused by unsafe abortion consist of; haemorrhage, infection, incomplete abortions, uterine perforation, and damage to the genital tract [9, 11–14].

As indicated by various studies, induced abortion is affected by a wide range of factors such as age [16], residence [8, 17], accommodation [7, 18], year of study [19, 20], substance use [17, 19, 21, 22], history of contraceptive use [22, 23], knowledge on abortion law [7], age first sex [23], and the number of sexual partners [22] are factors affecting induced abortion.

Advancing women’s access to safe and legal abortion is a priority for women’s reproductive health and rights, per the Sustainable Development Goals (SDGs) focused on health and gender equality [24]. For this reason, reducing induced abortion particularly unsafe abortion has caught the attention of numerous global and national organizations. The government of Ethiopia had revised the abortion law to reduce unsafe abortion and its complications [25]. Improving equitable access to quality reproductive services is also one of the key strategic objectives of the National Reproductive Health Strategy of Ethiopia (2016–2020) [26]. Moreover, a communication strategy on HIV/AIDS and sexual reproductive health was developed to reduce sexual and reproductive health problems of students of higher education institutions [27]. However, induced abortion has continued to be a major reproductive health problem in higher education institutions in Ethiopia. Knowing the magnitude of induced among students is critical for designing and realizing effective interventions to curb the problem. The studies done on induced abortion among female students in higher education institutions in Ethiopia found varied and inconclusive findings. The prevalence of induced abortion among female students in institutions of higher education in Ethiopia varied from 2.8% in Arba Minch town [7] to 18.8% in Mizan Tepi University [8]. Thus, this review aimed to estimate the pooled prevalence of induced abortion among female students in higher education institutions in Ethiopia.

## Methods

### Study design and search strategy

The Preferred Reporting Items for Systematic Review and Meta-Analysis (PRISMA) guideline was rigorously followed throughout this review [28] (S1 Table). A Measurement Tool to Assess Systematic Reviews (AMSTAR-2) was also used [29] (S2 Table). A systematic review and meta-analysis of published and unpublished studies were performed to assess the pooled prevalence of induced abortion among higher education institutions in Ethiopia. Electronic databases such as PubMed, Cochrane Library, Hinari, Google Scholar, CINAHL, and Global Health were used. The following key terms were used to search studies: "prevalence", "magnitude", "proportion", "incidence", "induced abortion", "abortion", "safe abortion", "unsafe abortion", "miscarriage", "legal abortion", “illegal abortion”, and “criminal abortion”, "factors", "determinants", "predictors", "factors associated", "associated factors", "risk factors", "University", "College", "Higher education institutions", "campus", "students", "undergraduate students", "female", "Ethiopia" by a combination of Boolean operators “AND” or “OR” as applicable and the search was made by two authors independently (BK and BA).

### Inclusion and exclusion criteria

This review includes all accessible studies done from January 1, 2010, to June 30, 2022. All published and unpublished studies conducted on the prevalence of induced abortion in higher education institutions in Ethiopia were incorporated in the review. All observational studies with English language publications that measured the prevalence of induced abortion among female students were considered in this review. But, studies with poor methodological quality were omitted from the review.

### Study selection, quality appraisal, and data extraction

All articles explored from selected databases were exported to Endnote X8 and duplicate files were dropped. Two investigators (YD and MA) screened the leftover articles and abstracts for inclusion in the full-text appraisal. The difference between reviewers was managed through discussion, and disagreement was handled by the third party (MY). The Joanna Briggs Institute (JBI) critical appraisal checklist for the prevalence study was used to evaluate the quality of articles that fulfilled the inclusion criteria [30]. Two reviewers independently assessed articles prior to inclusion in the review. Articles with quality scores of fifty and above were considered in the final review.

Microsoft Excel 2010 sheet was used for data extraction. The information on the author’s name, year of publication, year of study, study design, study area, response rate, sample size, study quality score, and prevalence were contained in the data extraction tool.

### Statistical methods and analysis

The analysis was carried out using STATA 14 software. Forest plots were used to present the prevalence of induced abortion among female students in higher education institutions in Ethiopia. The heterogeneity was assessed by using the I^2^ statistics, and it was declared using a p-value of less than 0.05 [31]. Subgroup analyses were performed by different study characteristics such as study year (before 2015 or 2015 and above), sample size (large or small), response rate (high or low), and study quality score (low or high score). The Egger regression asymmetry test was also used to check publication bias and confirmed with a p-value of less than 0.05 [32]. Moreover, Duval and Tweedie’s ‘trim and fill’ method was done to estimate the number of missing studies from the meta-analysis [33].

## Results

### Study selection

**Fig 1:**
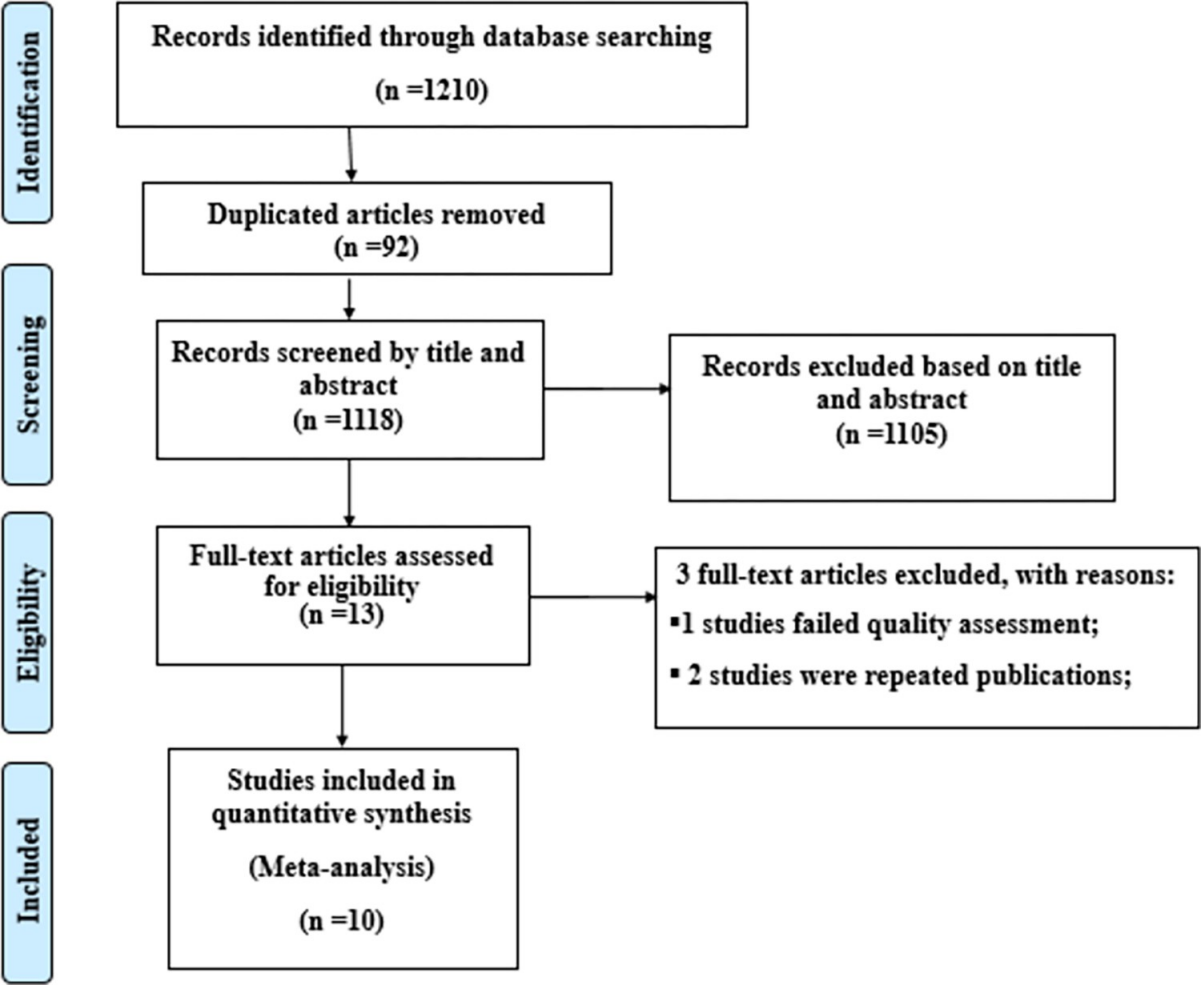
PRISMA flow diagram of the included studies for the systematic review and meta-analysis of induced abortion among female students at higher education institutions in Ethiopia.

This review included published and unpublished studies on induced abortion among female students at higher education institutions in Ethiopia. A total of 1210 records were identified through electronic database searching. Ninety-two duplicated records were removed, and the rest 1105 articles were excluded using their titles and abstracts. Thirteen full-text articles were evaluated for eligibility. From these, 3 full-text articles were omitted, and a total of ten articles were considered in the review (Fig 1).

### Characteristics of included studies

All studies included in this systematic review and meta-analysis were cross-sectional [7, 8, 16, 18–23, 34]. The sample size of the studies ranged from 213, a study done at Addis Ababa University [18] to 813, another study done in Arba Minch town [7]. Overall, this systematic review and meta-analysis included a total of 4656 study participants. The studies were carried out from 2011 to 2021 in different parts of Ethiopia (Table 1).

**Table 1:** Summary characteristics of studies included in the systematic review and meta-analysis.

Authors and Publication year	Study year	Study area	Study design	Sample size	Response rate	Prevalence	Quality score	Funding Source
Geda YF et al, 2020	2019	Hawassa University	Institutional based cross-sectional	741	95.7	9.58	78%	No
Biza N et al, 2018	2016	Samara University	Institutional based cross-sectional	509	93.2	8.84	56%	Not reported
Gelaye AA et al, 2014	2011	Wolaita Sodo University	Institutional based cross-sectional	493	93.0	6.23	78%	Wolaita Sodo University, UNICEF, UNFPA
Mitiku S et al, 2015	2015	Wachamo University	Institutional based cross-sectional	461	95.6	5.86	67%	Not reported
Animaw and Bogale, 2014	2011	Arba Minch Town	Institutional based cross-sectional	813	96.2	2.83	89%	Not reported
Beyene and Meshesha, 2020	2019	Dilla University	Institutional based cross-sectional	548	97.8	6.39	67%	Not reported
Thomas G et al, 2016	2013	Addis Ababa University	Institutional based cross-sectional	213	86.2	9.86	67%	Addis Ababa University
Romano and Bekele, 2020	2019	Jimma University	Institutional based cross-sectional	222	95.7	3.15	56%	Not reported
Nigussie T et al, 2020	2018	Mizan Tepi University	Institutional based cross-sectional	420	99.3	18.81	78%	No
Muche ZT, et al, 2021	2021	Debre Tabor Town	Institutional based cross-sectional	238	98.3	18.64	56%	Not reported

### Prevalence of induced abortion

**Fig 2:**
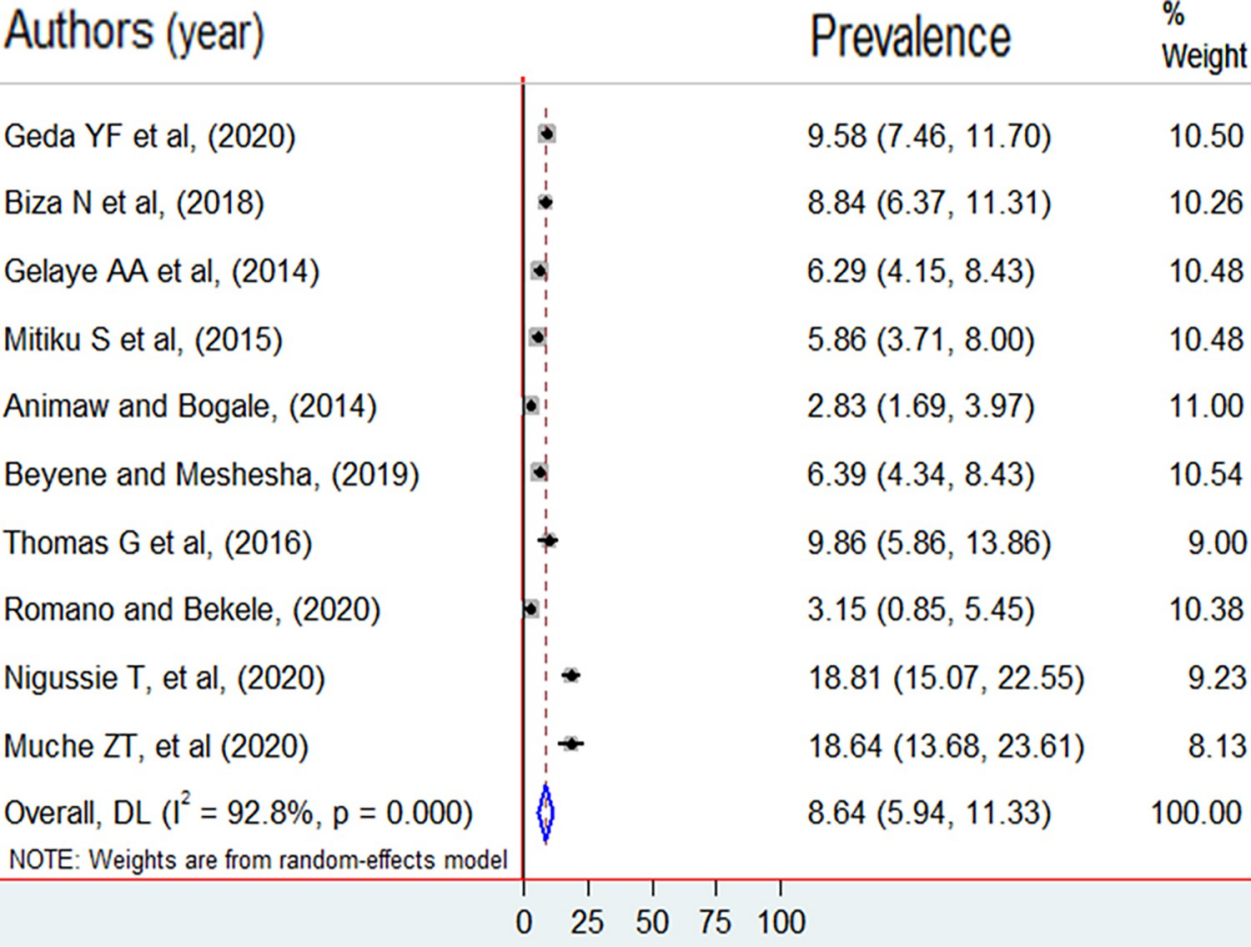
The prevalence of induced abortion among female students in higher education institutions in Ethiopia, 2011 to 2021.

**Fig 3:**
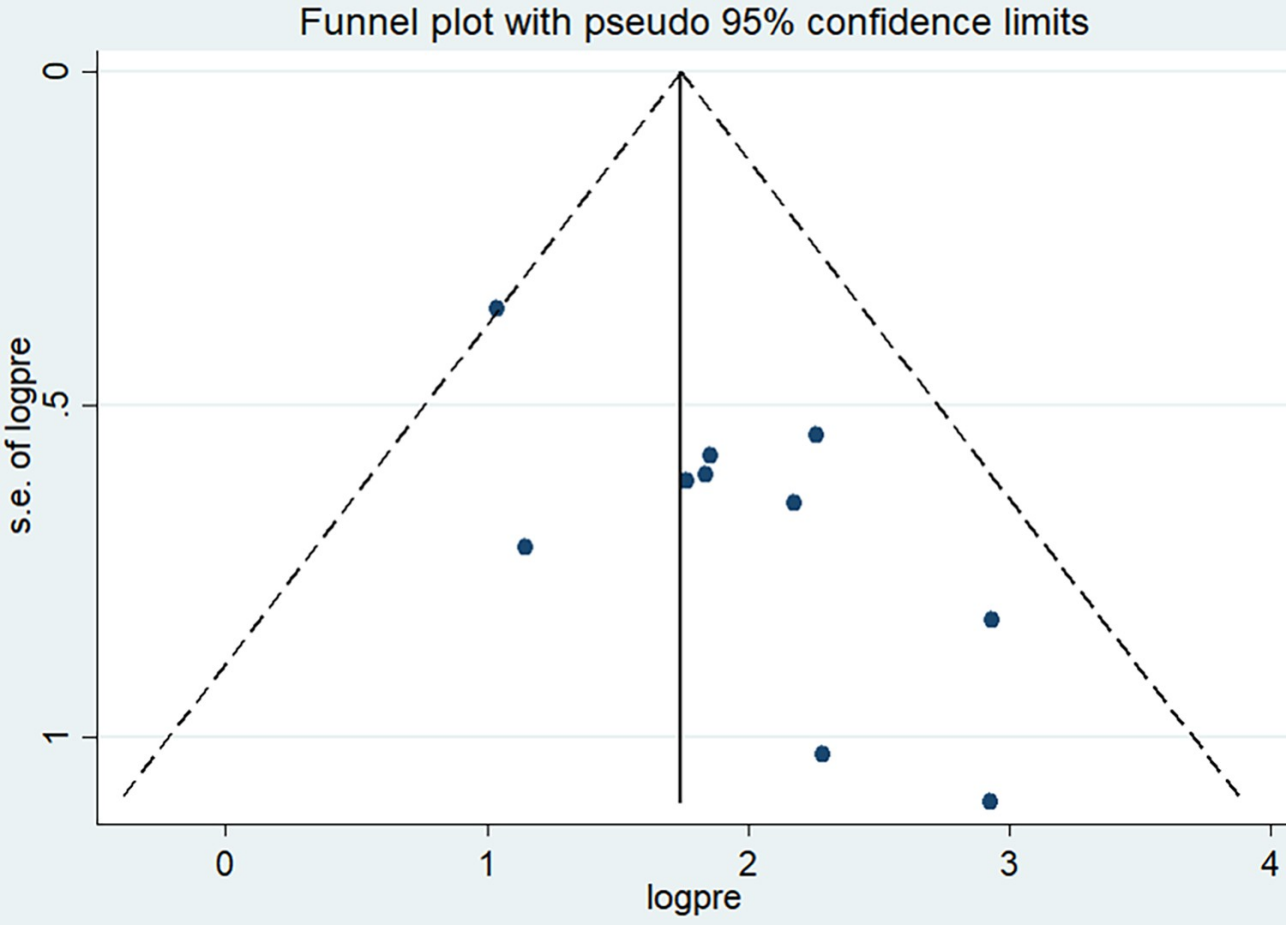
Funnel plot with 95% confidence limit of pooled prevalence of induced abortion among female students in higher education institutions of Ethiopia, 2011 to 2021.

**Fig 4:**
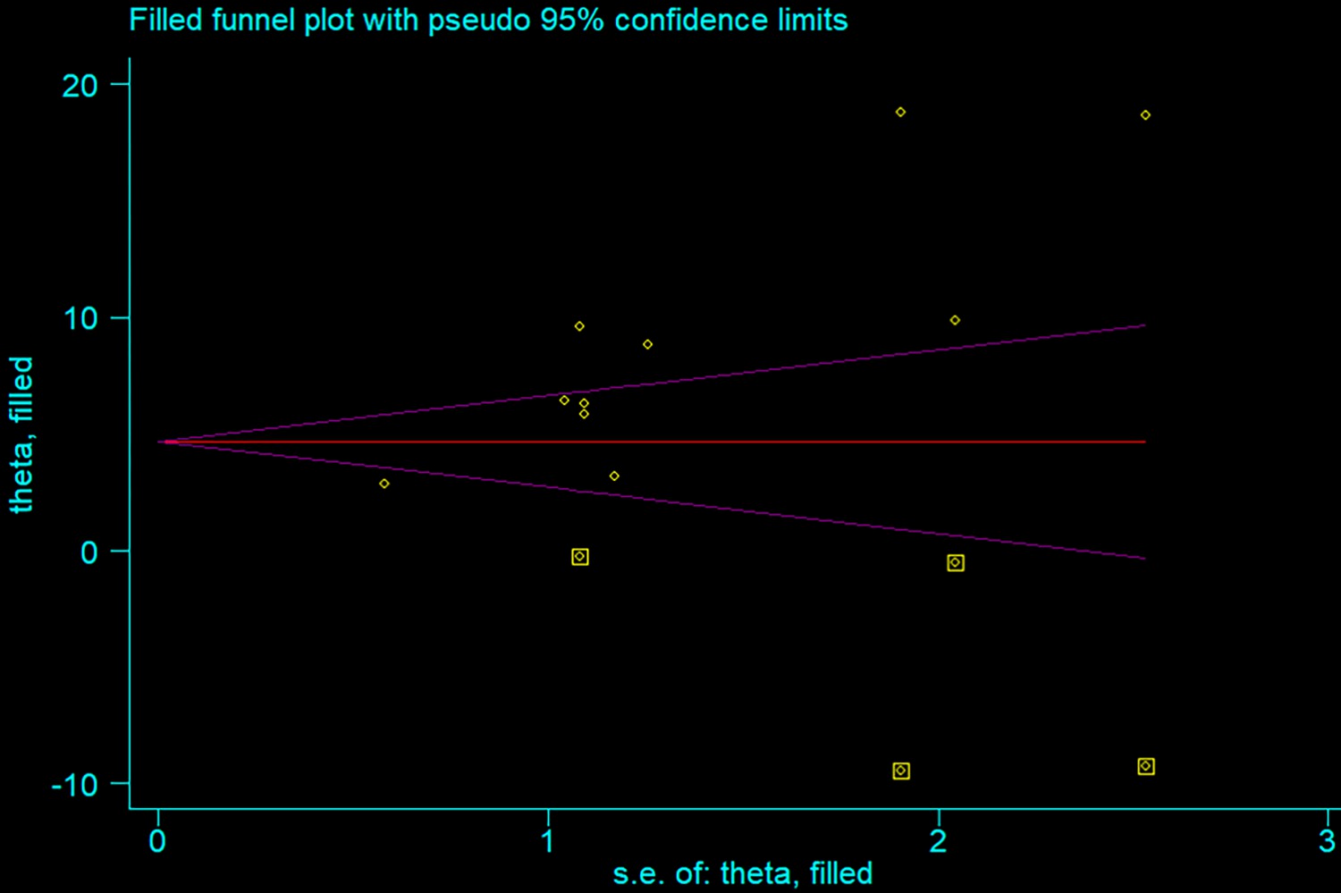
Funnel plot with 95% confidence limit of a simulated meta-analysis containing 14 studies.

The overall pooled prevalence of induced abortion among female students in higher education institutions in Ethiopia was 8.64% (95%CI: 5.94, 11.33). However, due to the presence of publication bias trim and fill method of analysis was done to correct for funnel plot asymmetry and adjust the final pooled estimate. Therefore, the final adjusted pooled prevalence of induced abortion was 5.06% (95%CI: 2.16, 7.96). The highest prevalence of induced abortion was 18.8% in a study done at Mizan Tepi University [8], and the lowest prevalence of induced abortion was reported in Arba Minch Town (2.8%) [7]. A significant heterogeneity was detected among included studies in the meta-analysis, I^2^ = 92.8%, p < 0.001 (Fig 2). The funnel plot also revealed an asymmetrical appearance (Fig 3). Furthermore, Egger’s regression asymmetry test indicated significant publication bias, p-value = 0.012. Thus, Duval and Tweedie’s ‘trim and fill’ method was performed to estimate the number of missing studies from the meta-analysis as a source of publication bias (Fig 4).

### Sub-group analysis

Subgroup analyses were done by different study level characteristics for instance study year, sample size, response rate, and study quality score to detect the source of heterogeneity. But, heterogeneity still exists. Therefore, the heterogeneity may be explained by other factors not considered in this review. The prevalence of induced abortion among studies conducted before 2015 was 5.93% (95%CI: 2.28, 9.58). The prevalence of induced abortion among studies with large and small sample size was 6.70% (95%CI: 3.91, 9.49) and 11.04% (95%CI: 5.15, 16.93), respectively (Table 2).

**Table 2:** Subgroup analysis of the prevalence of induced abortion at higher education institutions in Ethiopia, 2011–2021.

Subgroup	Number of studies	Total sample	Prevalence (95%CI)	Heterogeneity	
				I^2^	p-value
By sample size					
Large	5	3104	6.70 (3.91, 9.49)	90.8	< 0.001
Small	5	1552	11.04 (5.15, 16.93)	94.4	< 0.001
By study quality					
High	4	2976	9.15 (3.76, 14.54)	96.5	< 0.001
Low	6	1680	8.24 (5.25, 11.24)	86.6	< 0.001
By response rate					
High	7	3234	8.90 (5.27, 12.53)	94.8	< 0.001
Low	3	1422	7.97 (5.85, 10.08)	44.5	0.165
By study year					
Before 2015	3	1519	5.93 (2.28, 9.58)	88.0	< 0.001
2015 and above	7	3137	9.79 (6.37, 13.21)	92.1	< 0.001
Total	10	4656	8.64 (5.94, 11.33)	92.8	< 0.001

## Discussion

Induced abortion is a major public health issue and remains women’s reproductive health and human right concern globally. This systematic review and meta-analysis were conducted to estimate the prevalence of induced abortion among female students at higher education institutions in Ethiopia. The pooled prevalence of induced abortion among higher education institution female students in Ethiopia is 5.06% (95%CI: 2.16, 7.96) or 51 per 1000 female students.

This finding is in line with a systematic review and meta-analysis on induced abortion globally (58.1 per 1000 women) [35]. This finding is also comparable with the overall rate of abortion in Ethiopia (28 per 1000 women) [5], Sub-Saharan Africa (33 per 1000 women) [2], and globally (39 per 1000 women) [2]. This review considers only induced abortion and the majorities of female students were youths and not married. Female students in higher education institutions are expected to have fewer pregnancy and abortion rates as compared to women of reproductive age as a whole. However, this review finds a high rate of induced abortion which is similar to the national, regional, and global overall abortion rate. This might be due to almost all pregnancies of female students are unintended and end with induced abortion. Moreover, higher education institution students may have better access to abortion care services than other women of reproductive age. Additionally, lack of direct parental control, the need to enjoy their newly discovered freedom, sexual experimentation, lack of comprehensive knowledge on sexual and reproductive health problems, and high rate of sexual violence [36–38] may contribute to the high rate of induced abortion.

This finding is also in line with the magnitude of abortion among youths in Ethiopia (25 per 1000 women) [6]. However, this review does not include spontaneous abortion. Thus, this review finds a higher induced abortion rate. This could be due to more than one-third of youths included in 2016 EDHS were married and had fewer unintended pregnancies compared to female students in higher education institutions. Furthermore, nearly three-fourths of youths included in the 2016 EDHS were rural residents with various cultural and religious restrictions and poor access to abortion care services.

The level of induced abortion among female students in higher education institutions showed a modest increment. The level of induced abortion among studies conducted in the years 2015 to 2019 [8, 16, 20–23, 34] was higher than the studies conducted before 2015 [7, 18, 19]. This high rate of induced abortion among female students in higher education institutions implies that there was a high rate of unintended pregnancy. This calls for efforts to meet the need for sexual and reproductive health services including sexuality education, family planning, and abortion care services.

Lacks of access to quality abortion care threaten women’s physical and mental well-being and a range of human rights of women and girls. Thus, providing universal access to safe, affordable, timely, and respectful abortion care is an essential and cost-effective strategy for reducing maternal mortality and achieving targets 3.1, 3.7, and 5.6 [24]. Female students in higher education institutions, in particular, require universal access to youth-friendly and nonjudgmental sexual and reproductive health services.

The PRISMA guideline was rigorously followed at all steps of the systematic review and meta-analysis. However, most of the articles included in the review had a small sample size, which may affect the pooled estimate, decreases statistical power, and permits large standard errors. Besides, there were limited studies that presented factors associated with induced abortion. The factors assessed in those studies also differ across studies. Due to this, this review was unable to identify predictors of induced abortion among female students in higher education institutions.

## Conclusions

The rate of induced abortion among female students in higher education institutions in Ethiopia is high. The government and other concerned bodies should make an effort to reduce unintended pregnancy through the provision of youth-friendly and non-judgmental sexual and reproductive health services. Family planning and comprehensive abortion care services should be programming priorities for organizations working on the sexual and reproductive health of university students. Large-scale studies are needed to identify the determinants of induced abortion. Researchers should also investigate the magnitude of unsafe abortion among female students in higher education institutions.

## Supporting information

S1 Table PRISMA-P 2009 checklist. (DOC)

S2 Table AMSTAR 2: A critical appraisal tool for systematic reviews. (DOCX)

S1 File The search electronic strategy. (DOCX)

## Abbreviations

AOR: Adjusted Odds Ratio
CI: Confidence Interval
EDHS: Ethiopian Demographic and Health Survey
JBI: Joanna Briggs Institute
SDG: Sustainable Development Goal
UNFPA: United Nations Population Fund
UNICEF: United Nations Children’s Fund
