# Supplementary material for: Induced abortion among female students in higher education institutions in Ethiopia: A systematic review and meta-analysis
Source: PLoS One. 2023 Jan 20;18(1):e0280084. doi: 10.1371/journal.pone.0280084 (PMC9858066; doi:10.1371/journal.pone.0280084)
Supplement: S1 File — (DOCX) [file pone.0280084.s003.docx]

**S1 file. Searching strategy**

**PubMed**

((((((((((((((((((((((((((((Prevalence) OR (proportion)) OR (magnitude)) OR (incidence)) AND ("induced abortion")) OR (abortion)) OR ("safe abortion) ") AND ("unsafe abortion")) OR (miscarriage)) OR ("legal abortion ")) OR ("illegal abortion ")) OR ("criminal abortion ")) AND (factors)) OR (determinants)) OR (predictors)) OR ("factors associated")) OR ("associated factors")) OR ("risk factors")) AND (University)) AND (College)) OR ("Higher education institutions")) OR (campus)) AND (students)) OR ("undergraduate students")) AND (Female)) AND (Ethiopia)

Filter applied: Specie-Humans, Language-English, Sex-Female, Age- (Adolescent: 13-18 years, Adult: 19-44 years), and year- (January 1, 2010- June 30, 2022).

**CINHAL**

((((((((((((((((((((((((((((Prevalence) OR (proportion)) OR (magnitude)) OR (incidence)) AND ("induced abortion")) OR (abortion)) OR ("safe abortion) ") AND ("unsafe abortion")) OR (miscarriage)) OR ("legal abortion ")) OR ("illegal abortion ")) OR ("criminal abortion ")) AND (factors)) OR (determinants)) OR (predictors)) OR ("factors associated")) OR ("associated factors")) OR ("risk factors")) AND (University)) AND (College)) OR ("Higher education institutions")) OR (campus)) AND (students)) OR ("undergraduate students")) AND (Female)) AND (Ethiopia)

Refined by: Year- January, 2010- June 2022; Age- (13-18 years, 19-44 years); Journal subset- (Public health, Health promotion/education)

The search was made from 5-15 July 2022.
